# Cortisol levels and neuropsychiatric diagnosis as markers of postoperative delirium: a prospective cohort study

**DOI:** 10.1186/cc12548

**Published:** 2013-03-01

**Authors:** Jakub Kazmierski, Andrzej Banys, Joanna Latek, Julius Bourke, Ryszard Jaszewski

**Affiliations:** 1 Department of Old Age Psychiatry and Psychotic Disorders, Medical University of Lodz, Czechoslowacka 8/10, 92-216 Lodz, Poland; 2 Department of Anaesthesiology and Intensive Cardiologic Care, 1st Chair of Cardiology and Cardiac Surgery, Medical University of Lodz, Sterlinga 1/3, 91-425 Lodz, Poland; 3 Central Veterans Hospital, Sterlinga 1/3, 91-425 Lodz, Poland; 4 The Centre for Psychiatry at The Wolfson Institute for Preventive Medicine, Barts and The London School of Medicine and Dentistry, Queen Mary University of London, 3rd floor Dominion House, 59 Bartholomew Close, London EC1A 7E, UK; 5 Department of Cardiac Surgery, 1st Chair of Cardiology and Cardiac Surgery, Medical University of Lodz, Sterlinga 1/3, 91-425 Lodz, Poland

## Abstract

**Introduction:**

The pathophysiology of delirium after cardiac surgery is largely unknown. The purpose of this study was to investigate whether increased concentration of preoperative and postoperative plasma cortisol predicts the development of delirium after coronary artery bypass graft surgery. A second aim was to assess whether the association between cortisol and delirium is stress related or mediated by other pathologies, such as major depressive disorder (MDD) or cognitive impairment.

**Methods:**

The patients were examined 1 day preoperatively with the Mini International Neuropsychiatric Interview and the Montreal Cognitive Assessment and the Trail Making Test to screen for depression and for cognitive impairment, respectively. Blood samples for cortisol levels were collected both preoperatively and postoperatively. The Confusion Assessment Method for the Intensive Care Unit was used within the first 5 days postoperatively to screen for a diagnosis of delirium.

**Results:**

Postoperative delirium developed in 36% (41 of 113) of participants. Multivariate logistic regression analysis revealed two groups independently associated with an increased risk of developing delirium: those with preoperatively raised cortisol levels; and those with a preoperative diagnosis of MDD associated with raised levels of cortisol postoperatively. According to receiver operating characteristic analysis, the most optimal cutoff values of the preoperative and postoperative cortisol concentration that predict the development of delirium were 353.55 nmol/l and 994.10 nmol/l, respectively.

**Conclusion:**

Raised perioperative plasma cortisol concentrations are associated with delirium after coronary artery bypass graft surgery. This may be an important pathophysiological consideration in the increased risk of postoperative delirium seen in patients with a preoperative diagnosis of MDD.

## Introduction

Coronary artery disease is the single largest cause of death in developed countries, and one of the leading contributors to death in the developing world [1,2]. Coronary artery bypass graft (CABG) surgery is a lifesaving treatment for severe ischemic heart disease. However, this procedure is associated with neuropsychiatric complications. These complications include delirium, which substantially worsens postoperative recovery and prognosis [3,4].

According to recent studies, the most prominent factors contributing to postoperative delirium include comorbid load (atrial fibrillation, prior stroke, anemia, peripheral vascular disease) as well as psychiatric comorbidity such as cognitive impairment and preoperative major depressive disorder (MDD) [5-7]. The pathological association between MDD and postoperative delirium is unclear. These disorders have been proposed to be linked by a greater rise in plasma cortisol, interleukins and abnormalities in amino acids [5,7,8]. However, few studies have attempted to or been able to identify the pathogenesis of delirium following cardiac interventions, although two recent important studies suggest an association with raised postoperative cortisol levels [9,10], whilst Plaschke and colleagues have additionally implicated increased levels of IL-6 [10]. These authors hypothesize that the increased cortisol level is a stress marker. However, although current thinking implicates cortisol and cytokine abnormalities in both MDD and cognitive impairment, neither of these was screened for in the studies cited above [9,10]. As such, the precise delineation as to whether this was related to surgical stress rather than additional neuropsychiatric comorbidities remains unclear. The failure to assess for comorbidities such as MDD, cognitive impairment and impaired executive function may therefore represent a confound in the accurate interpretation of prior studies.

In light of this, the primary objective of the current study was to investigate the association between preoperative and postoperative plasma cortisol concentrations and the development of postoperative delirium. The secondary objective was to assess whether any association between cortisol and delirium is stress related or mediated by way of MDD or cognitive impairment. We hypothesized that: delirium after CABG surgery is independently associated with increased preoperative cortisol levels; these raised cortisol levels may be related to pre-existing conditions, such as MDD, cognitive disturbances and aging; increased reactivity of the hypothalamus-pituitary-adrenal (HPA) axis associated with MDD results in a greater cortisol response postoperatively as compared with patients without MDD; and patients with MDD are at a greater risk of delirium postoperatively as a consequence of these mechanisms.

## Materials and methods

### Overview

The study was approved by the Ethics Committee of the Medical University of Lodz, Poland and was performed in accordance with the ethical standards of the Declaration of Helsinki. The study was conducted in the 14-bed cardiac surgical intensive care unit (ICU) of a university teaching hospital (University Hospital, Central Veterans Hospital, Poland) between May and September 2011. The subjects signed an informed consent the day before their operation. The inclusion criteria were: consecutive adult patients scheduled for CABG surgery with cardiopulmonary bypass. The exclusion criteria were as follows: concomitant surgery other than CABG; history of adrenal gland disease; history of glucocorticoid therapy within the last year; non-Polish-speaking subjects; illiteracy; and patients with pronounced hearing and/or visual impairment.

### Preoperative psychiatric and psychological procedures

The study population was examined by a psychiatrist (JK) on the day prior to the scheduled operation using the Montreal Cognitive Assessment (MoCA) and the Trail Making Test Part B (TMT-B) to assess global cognition, and executive functions, respectively. The Mini International Neuropsychiatric Interview was additionally employed to assess for a diagnosis of MDD.

The MoCA was designed as a rapid screening instrument for mild cognitive dysfunction. This instrument assesses different cognitive domains: attention and concentration, executive functions, memory, language, visuoconstructional skills, conceptual thinking, calculations, and orientation [11]. The TMT-B is a widely used paper-and-pencil task that evaluates the executive functions and cognitive flexibility [12]. The Mini International Neuropsychiatric Interview is a structured diagnostic interview, developed jointly by psychiatrists and clinicians in the United States and Europe for *The Diagnostic and Statistical Manual of Mental Disorders*, Fourth Edition and for International Classification of Diseases, Tenth Revision psychiatric disorders [13].

### Anesthesia and surgery

For premedication, midazolam 7.5 mg per orally 1 hour before surgery was used. Before inducing anesthesia in the patients, routine monitoring was installed: electrocardiography leads II and V5, invasive radial arterial blood pressure monitoring, central venous pressure monitoring, cerebral oxygen saturation, and peripheral oxygen saturation. A standard anesthesia technique was used for all patients. Induction of anesthesia involved fentanyl 5 to 10 μg/kg, midazolam 0.1 to 0.15 mg/kg, and rocuronium 0.6 to 0.8 mg/kg. Medication during maintenance was as follows: fentanyl in continuous intravenous infusion of dose 1 to 2 μg/kg, midazolam (0.1 to 0.2 mg/kg), and interrupted doses of rocuronium. Ventilation was provided with a breathing mixture of FiO_2 _0.5 and air to maintain end-tidal carbon dioxide at 35 mmHg. From surgical incision to cardiopulmonary bypass connection, sevoflurane 0.5 to 1.5 vol.% was used. Intraoperative monitoring additionally included end-tidal expiratory carbon dioxide, nasopharyngeal temperature, bladder temperature, and urine output. Pulmonary artery catheter was inserted when necessary. In cases of hypotension, norepinephrine was employed to counteract profound vasodilatation, at a rate 0.05 to 1 μg/kg/minute, to maintain mean arterial pressure above 60 mmHg.

All patients underwent CABG surgery through a median sternotomy. During the study period, the surgical and cardiopulmonary bypass procedures remained similar. The patients were operated on under normothermia using antegrade cold crystalloid St Thomas' Hospital cardioplegic solution No. 2 (4 to 6°C). After surgery, all patients were transferred to the ICU and were placed on mechanical ventilation. Until extubation, 102 (90%) study patients were sedated with midazolam in continuous infusion of 0.075 to 0.2 mg/kg/hour, plus additional interrupted doses of 0.1 to 0.2 mg/kg morphine, while the remaining participants were sedated with propofol perfusion at a rate of 1 to 2 mg/kg/hour, targeting Ramsay Sedation Scale scores of -4 to -5. The acceptable levels of arterial blood gases (maintained at pH 7.35 to 7.45, partial pressure of carbon dioxide in arterial blood 35 to 45 mmHg, partial pressure of oxygen in arterial blood > 90 mmHg) and oxygen saturations > 90% were necessary criteria for extubation. Additionally, patients were required to be awake and cooperative, hemodynamically stable with a body temperature > 36.5°C (preferably normothermic), have no active bleeding (< 400 ml/2 hours) nor coagulopathy and to demonstrate a return of muscle strength (defined by > 5 seconds head lift/strong hand grip). As a standard, weaning from mechanical ventilation and extubation in uncomplicated cases took place 4 to 6 hours after the operation.

Intraoperative and postoperative measures were recorded on the basis of local protocols pertaining to postoperative management of patients on the cardiac ICU. The lowest intraoperative hemoglobin concentration was entered into current analysis. During the surgery and postoperatively, the presence of atrial fibrillation was recorded by 24-hour electrocardiography monitoring. One-time and multiple increases of partial pressure of carbon dioxide in arterial blood ≥ 45 mmHg and a drop of partial pressure of oxygen in arterial blood ≤ 50 mmHg were recorded and entered into the analysis.

### Measurement of serum cortisol and IL-2 levels

The venous blood samples were taken twice during the study period: the day prior to the surgery (baseline measurement) and on the first postoperative day, between the hours 08:00 and 09:00 a.m. The blood samples were centrifuged at 7,000 rpm for 10 minutes and were refrigerated for a maximum of 1 month at -20°C until biochemical parameters were determined. The serum cortisol concentration was measured with a competitive electrochemiluminescent enzyme immunoassay in a calibrated Elecsys 2010 analyzer (Roche Diagnostic GmbH, Mannheim, Germany). The normal range of plasma cortisol according to the laboratory where measurements were performed is 171 to 536 nmol/l in the morning and 63 to 327 nmol/l in the evening. The venous blood samples for IL-2 were taken on the first day postoperatively, between the hours of 08:00 and 09:00 a.m. The serum IL-2 concentration was determined by chemiluminescent immunoassay technology. The normal concentration of plasma IL-2 according to the laboratory where the measurement was performed is < 710 U/ml. The tests were conducted by investigators that were blinded to clinical data.

### Delirium diagnosis

None of the patients had preoperative delirium while being assessed according to the Confusion Assessment Method. Following surgical interventions, the Confusion Assessment Method for the Intensive Care Unit was used to diagnose delirium [14]. Each individual was assessed by one of the study psychiatrists twice a day (from 08:00 to 10:00 a.m. and from 08:00 to 10:00 p.m.) within the first 5 days after surgery. Before each administration of the Confusion Assessment Method for the Intensive Care Unit, the level of sedation/arousal was assessed using the Richmond Agitation Sedation Scale [15]. If the patient was deeply sedated or was unarousable (-4 or -5 on the Richmond Agitation Sedation Scale), evaluation was stopped and repeated later. If the Richmond Agitation Sedation Scale was above -4 (-3 through +4), assessment with the Confusion Assessment Method for the Intensive Care Unit was administered.

### Statistical analysis

Quantitative variables are expressed as medians and interquartile ranges (IQRs). For categorical variables, the number of observations (*n*) and fraction (%) were calculated. Normality was tested using the Shapiro-Wilk's test for normality. Differences between two independent samples for continuous data were analyzed using the Mann-Whitney U test (since the distributions of variables were different from normal).

For categorical variables, statistical analysis was based on the chi-squared test or the chi-squared test with Yates' adjustment. Spearman's rank correlation coefficients were calculated to assess the correlation between two quantitative variables. The minimum study sample size was calculated using the power analysis, estimating the expected effects from the pilot data and assuming an alpha level of 0.10 and a power of 80% (minimum sample size for each group is 37 patients).

Distributions for postoperative cortisol levels were different from normal in both depression and nondepression groups (*P *< 0.001). Similarly, the assumption of homogeneity of variance was not satisfied for postoperative cortisol levels (*P *< 0.01). The nonparametric Friedman's version of analysis of variance was thus used to compare cortisol before and after CABG surgery considering depression. Initially, baseline and perioperative variables were evaluated for univariate association with postoperative delirium. For quantitative variables (preoperative and postoperative cortisol concentration), significantly associated with the occurrence of delirium, receiver operating characteristic curves were drawn and decision thresholds were found. The sensitivity, specificity, positive predictive value and negative predictive value were calculated. Odds ratios with 95% confidence intervals and standard errors were also presented. Factors significant in univariate comparisons (*P *< 0.10) were included in a forward stepwise logistic regression model to identify the set of the independent risk factors for delirium. The results were considered significant for *P *< 0.05. All of the calculations were performed using STATISTICA (version 9, 2009; StatSoft, Inc., Tulsa, OK, USA) and SPSS (SPSS Statistics, version 19; IBM, Armonk, NY, USA) software.

## Results

**Figure 1:**
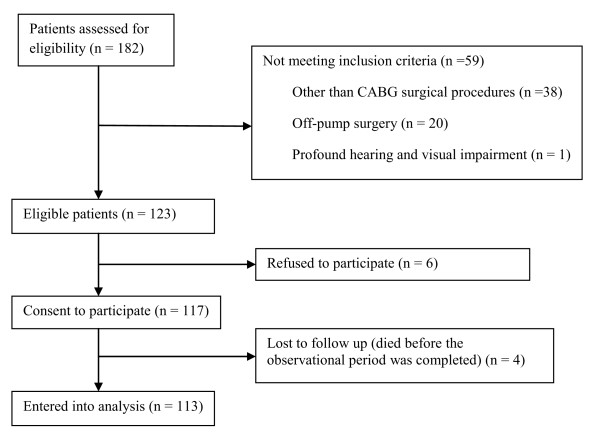
**Number of patients excluded and included in the data analysis**. CABG, coronary artery bypass graft.

One hundred and eighty-two patients underwent CABG surgery during the study period; of these, 59 subjects did not meet the inclusion criteria (Figure 1). Baseline demographic characteristics and patients' comorbidities are presented in Table 1. Postoperative delirium developed in 36% (41 of 113) of patients. The median duration of delirium was 3.5 days (IQR = 2 to 4). The frequency of diagnosis of delirium decreased with an increasing number of postoperative days (day 1, *n *= 22, 54%; day 2, *n *= 13, 32%; day 3, *n *= 4, 10%; day 4, *n *= 1, 2%; day 5, *n *= 1, 2%). Patients with postoperative delirium had a significantly longer stay in the ICU (6 vs. 2 days; *P *< 0.0001) and a longer total duration of hospitalization (19 vs. 11 days; *P *< 0.0001) compared with patients who did not develop delirium.

**Table 1:** Demographic characteristics and comorbidities of all 113 patients enrolled in the study

Characteristic	*n*	%
Demographics		
Age^a^	64	(59 to 71)
Gender male	90	79.65
Years of education		
Between 1 and 7 years	31	28
Between 8 and 11 years	66	58
12 years or more	16	14
Living area		
City > 100,000 people	54	48
City < 100,00 people	33	29
Country	26	23
Social status		
Living with family	100	88.5
Living alone	13	11.5
Psychiatric comorbidities		
Depression	18	16
TMT-B score^a^	130	(96 to 200)
MoCA score^a^	26	(24 to 27)
MoCA score < 25	36	32
Physical comorbidities		
Anemia (hemoglobin < 10 mg/dl)	18	16
Urea concentration > 7 mmol/l	33	29.2
Creatinine concentration > 120 μmol/l	7	6.2
Peripheral vascular disease	23	20.3
Atrial fibrillation	9	8
Arterial hypertension	94	83
Diabetes	39	35
Cerebrovascular disease	6	5.3
New York Heart Association grade		
0	13	11
I	30	27
II	52	46
III	18	16
IV	-	
Canadian Cardiovascular Society degree		
0	4	4
I	8	7
II	48	42
III	50	44
IV	3	3

MoCA, Montreal Cognitive Assessment; TMT-B, Trail Making Test Part B. ^a^For continuous variables, the median and interquartile range is given.

The results of the univariate analysis of variables related to the condition of participants, anesthesia and surgical procedures are shown in Tables 2, 3, and 4. The unadjusted risk of postoperative delirium was higher both for patients with increased preoperative and postoperative cortisol concentrations (odds ratio = 1.004, *P *= 0.006; odds ratio = 1.002, *P *< 0.0001, respectively). Subjects with higher preoperative and postoperative cortisol level remained at increased risk of developing delirium after controlling for the following variables significant in univariable analysis: age, gender, cognitive performance (MoCA and TMT-B scores), preoperative urea, creatinine, hemoglobin concentration, peripheral vascular disease, duration of surgery, dose of midazolam, intraoperative hemoglobin level, partial pressure of oxygen, partial pressure of carbon dioxide, atrial fibrillation, and IL-2 concentration. However, after controlling for preoperative depression, only preoperative cortisol concentration remained significant, irrespective of the cortisol level after surgery (Table 5).

**Table 2:** Biomarkers and variables related to demography and mental condition of patients analyzed in univariate analysis

Variable	Nondelirious (*n *= 72)	Delirious (*n *= 41)	Odds ratio (95% CI)	*P *value
Age (years)	61.5 (58 to 67.5)	68.8 (64 to 74)	1.13 (1.07 to 1.20)	< 0.0001
Gender female	11 (15.28%)	12 (29.27%)	2.29 (0.92 to 5.74)	0.076
MoCA score	26 (25 to 27)	25 (23 to 26)	0.82 (0.70 to 0.94)	0.0001
TMT-B score	100 (90 to 161.5)	210 (145 to 300)	1.01 (1.00 to 1.02)	< 0.0001
Depression	2 (2.78%)	16 (39.02%)	22.40 (6.72 to 74.65)	< 0.0001
Preoperative cortisol (nmol/l)	316.5 (239.6 to 423)	444.8 (288.7 to 528.2)	1.004 (1.001 to 1.006)	0.006
Postoperative cortisol (nmol/l)	876.3 (672.1 to 1,101)	1,162 (910 to 1,505)	1.002 (1.001 to 1.003)	< 0.0001
Postoperative IL-2 (U/ml)	721.5 (569.5 to 1,043)	1,179 (875 to 1,414)	1.002 (1.001 to 1.003)	< 0.0001

Data presented as *n *(%); for continuous variables the median and interquartile range is given. CI, confidence interval; MoCA, Montreal Cognitive Assessment; TMT-B, Trail Making Test Part B.

**Table 3:** Variables related to physical condition of patients analyzed in univariate analysis

Variable	Nondelirious (*n *= 72)	Delirious (*n *= 41)	Odds ratio (95% CI)	*P *value
Peripheral vascular disease^a^	11 (15.28%)	12 (29.27%)	2.29 (0.92 to 5.74)	0.076
Urea concentration (mmol/l)^a^	5.6 (4.9 to 7.15)	6.5 (5.5 to 7.7)	1.19 (1.02 to 1.39)	0.008
Creatinine concentration (μmol/l)^a^	74 (62.5 to 90)	78.5 (70 to 99.5)	1.01 (0.99 to 1.03)	0.041
Anemia^a, b^	7 (9.72%)	11 (26.83%)	3.40 (1.25 to 9.30)	0.017
Atrial fibrillation^c^	3 (4.17%)	12 (29.3%)	5.70 (2.13 to 15.31)	0.001
Cerebrovascular disease^a^	2 (2.78%)	4 (9.76%)	3.78 (0.73 to 19.50)	0.112
Arterial hypertension^a^	59 (81.94%)	94 (85.37%)	1.29 (0.45 to 3.68)	0.640
Diabetes^a^	24 (33.33%)	15 (36.59%)	1.15 (0.52 to 2.57)	0.727
NYHA grade ≥ 3^a^	11 (15.28%)	7 (17.07%)	1.14 (0.41 to 3.22)	0.802
CCS degree ≥ 3^a^	30 (41.67%)	23 (56.10%)	1.79 (0.83 to 3.87)	0.139

Data presented as *n *(%); for continuous variables the median and interquartile range is given. CCS, Canadian Cardiovascular Society; CI, confidence interval; NYHA, New York Heart Association. ^a^Preoperative variable. ^b^Hemoglobin concentration < 10 mg/dl. ^c^Postoperative variable.

**Table 4:** Variables related to anesthesia and surgery analyzed in univariate analysis

Variable	Nondelirious (*n *= 72)	Delirious (*n *= 41)	Odds ratio (95% CI)	*P *value
Dose of midazolam during surgery (mg)	46.2 (35 to 50)	50 (45 to 50)	1.04 (1.01 to 1.08)	0.011
Duration of surgery (hours)	3 (2.5 to 3.5)	3.5 (3 to 4)	1.06 (0.83 to 1.35)	0.051
Hemoglobin concentration^a, b ^(mg/dl)	8.9 (7.8 to 10.7)	7.9 (6.5 to 8.6)	0.66 (0.53 to 0.84)	0.0001
PaCO2 ≥ 45^c, d ^(mmHg)	17 (23.6%)	19 (46.3%)	2.79 (1.25 to 6.27)	0.013
PaO2 ≤ 60^c, d ^(mmHg)	13 (18.06%)	25 (60.98%)	7.09 (3.10 to 16.21)	< 0.0001
Aortic cross-clamping^a ^(minutes)	485 (415 to 622)	519 (435 to 675)	1.01 (0.99 to 1.02)	0.100

Data presented as *n *(%); for continuous variables the median and interquartile range is given. CI, confidence interval. ^a^Intraoperative variable. ^b^The lowest intraoperative hemoglobin concentration was recorded and entered into the analysis. ^c^Postoperative variable. ^d^Both the one-time and multiple or sustained increase of partial pressure of carbon dioxide in arterial blood (PaCO_2_) ≥ 45 mmHg and the drop of partial pressure of oxygen in arterial blood (PaO_2_) ≤ 50 mmHg were recorded and entered into the analysis.

**Table 5:** Factors independently associated with delirium after CABG surgery revealed in multivariate stepwise logistic regression analysis^a^

Variable	Coefficient	Standard error	Odds ratio (95% CI)	*P *value
TMT-B^b^	0.016	0.004	1.02 (1.01 to 1.03)	< 0.0001
Creatinine concentration^b^	0.015	0.012	1.02 (0.99 to 1.04)	0.191
Dose of midazolam	0.081	0.028	1.08 (1.03 to 1.15)	0.005
Preoperative cortisol	0.005	0.002	1.005 (1.001 to 1.009)	0.025
Depression^b^	2.389	0.954	10.90 (1.68 to 70.67)	0.012
IL-2 concentration^c^	0.002	0.001	1.002 (1.001 to 1.004)	0.004
Constant	-12.964	2.725	-	< 0.0001

CI, confidence interval; TMT-B, Trial Making Test. ^a^The regression model is statistically significant: χ^2 ^= 76.889; *P *< 0.001. ^b^Preoperative variable. ^c^Postoperative variable.

According to receiver operating characteristic analysis, the most optimal cutoff values that predict the development of delirium were as follows: preoperative cortisol concentration ≥ 353.55 nmol/l, with sensitivity of 65.85% and specificity of 63.89%, positive predictive value of 50.94% and negative predictive value of 76.67% (odds ratio = 3.41) (area under the curve = 0.66; 95% confidence interval = 0.55 to 0.76; standard error = 0.05); and postoperative cortisol concentration ≥ 994.10 nmol/l, with sensitivity of 65.85% and specificity of 69.44%, positive predictive value of 55.10% and negative predictive value of 78.13% (odds ratio = 4.38) (area under the curve = 0.72; 95% confidence interval = 0.63 to 0.82; standard error = 0.05).

The median preoperative and postoperative cortisol concentrations in the whole population were 335.6 nmol/l (IQR = 247.5 to 459.5) and 940.7 nmol/l (IQR = 783.8 to 1,273), respectively. According to the Mann-Whitney U test, the median preoperative cortisol concentration was higher in patients with depression than in nondepression subjects: 483.1 nmol/l (IQR = 388.4 to 612.3) versus 318.8 nmol/l (IQR = 235.5 to 439.8) (*P *= 0.001), respectively. Similarly, the median postoperative cortisol concentration was higher in patients with depression compared with the nondepression group: 1,194.5 nmol/l (IQR = 936 to 1438) versus 908.4 nmol/l (IQR = 709 to 1,256) (*P *= 0.009), respectively. However, according to nonparametric analysis of variance, the interaction between the presence of depression and preoperative and postoperative cortisol concentration was not statistically significant (*P *= 0.447). This suggests that the postoperative cortisol concentration was higher than the preoperative, regardless of depression occurrence. The Spearman's rank correlation coefficients between preoperative cortisol and MoCA scores and between postoperative cortisol and MoCA scores were -0.21 (*P *= 0.025) and -0.14 (*P *= 0.130), respectively. The Spearman's rank correlation coefficients between preoperative cortisol and age and between postoperative cortisol and age were 0.18 (*P *= 0.049) and 0.25 (*P *= 0.007), respectively.

## Discussion

This study investigated the impact of increased preoperative and postoperative cortisol concentration in relation to a diagnosis of preoperative MDD and cognitive impairment on the risk of developing postoperative delirium.

Among 113 patients undergoing CABG, 36% (41) developed delirium. The effect of both preoperative and postoperative cortisol concentration on the risk of developing delirium was significant after controlling for demographic, physical, cognitive, surgical and anesthetic-related factors. However, when cortisol levels were controlled for MDD, only the preoperative cortisol concentration remained significant. The final multivariate regression analysis revealed that the preoperative cortisol level, MDD, impaired executive functions, higher creatinine and IL-2 concentrations and a higher dosage of midazolam independently increase the risk of postoperative delirium.

The incidence of postoperative delirium reported in the present study is in line with findings of similar contributions related to cardiac surgery (the reported estimates vary from 3 to 50%) [16-18]. In our previous study conducted in cardiac surgery patients [19], however, the incidence of delirium was lower (11.5%) compared with those currently reported (36%). This discrepancy may be due to differences in the groups studied, diagnostic approaches and the assessment tools used. In the first contribution, *The Diagnostic and Statistical Manual of Mental Disorders *Fourth Edition criteria were used to diagnose delirium and the participants were younger (mean age 62 years). Moreover, screening for delirium was conducted once a day starting from the second postoperative day. All of these factors may have affected the final estimates.

In the current study, the incidence of MDD and cognitive impairment (MoCA score < 25) were 16% and 32%, respectively. This is higher than the prevalence of unipolar depression in the general population (5 to 9% for females, 2 to 3% for males) and is consistent with the prevalence of MDD in CABG patients reported in other studies (15 to 20%) [20-22]. Recent studies confirmed that cognitive impairment is common among older patients undergoing major surgery, including cardiac interventions [5,6,18,23]. Depending on the diagnostic measures employed, the prevalence of cognitive disturbances ranges between 17 and 44%, and this is consistent with the results presented here (32%) [5,6,18]. Worthy of notice is that our previous study revealed cognitive disturbances in 100 out of 563 patients (17%). In the current study, however, we used more sensitive diagnostic instruments, which additionally enabled us to diagnose milder forms of cognitive impairment [11].

A number of studies have investigated risk factors for postoperative delirium, but findings have been heterogeneous. This inconsistency of the results may be, in part, related to the multifactorial etiology of delirium. Unfortunately, the pathophysiology and biological processes underlying this neuropsychiatric syndrome are poorly described - but it is possible that different mechanisms involved in delirium act through the same final pathways. This might explain the heterogeneity of research findings.

The most consistently reported independent associations with delirium in recent studies of cardiac surgery to date include older age, preoperative cognitive impairment and MDD [5,6,18]. MDD and advanced age may contribute to delirium through the elevated level of cortisol, secondary to activation of the limbic HPA axis in such individuals [7]. In this model, glucocorticoids inhibit endothelial cell proliferation and turnover in the hippocampus and prefrontal cortex [24], whilst HPA axis dysregulation results in decreased hippocampus volume [25]. Two recent studies investigated the association between plasma cortisol and delirium among cardiac surgery patients [9,10]. Plaschke and colleagues reported the association between increased cortisol concentration and delirium among a heterogeneous population of cardiac surgery patients in a univariate analysis that did not control for other factors - as such, the association with and significance of raised cortisol levels were not determined [10]. Mu and colleagues showed an independent association between elevated cortisol levels and postoperative delirium in individuals who underwent CABG surgery [9]. Both of these groups propose that increased cortisol concentration may be a marker of stress response, with the caveat that surgery-related stress is probably not the only factor contributing to elevated cortisol levels. Neither group was able to determine whether hypercortisolemia was a cause or an effect of postoperative delirium in the absence of baseline cortisol measurements, samples only being collected postoperatively. Furthermore, preoperative screening for potentially confounding neuropsychiatric disorders that were associated with altered cortisol levels and with delirium were not performed.

According to the results of present study, major depression prior to surgery is strongly and independently associated with an increased risk of postoperative delirium. Interestingly, high postoperative cortisol level also increases the risk of delirium, but this association lost significance once preoperative MDD was controlled for. Moreover, according to univariate analysis, the concentration of cortisol after surgery is significantly higher among patients suffering from depression when compared with nondepression subjects. These data suggest that, regarding delirium, depression is the primary factor affecting the condition of the ICU patients. Hypercortisolemia may be the factor that mediates the impact of MDD on postoperative cognition. This interpretation should be treated with caution, however, since the postoperative cortisol concentration was higher than the preoperative one regardless of depression occurrence, according to analysis of variance. On the contrary, our analysis revealed that a higher cortisol concentration measured the day prior to surgery independently increases the risk of delirium, even after controlling for depression, cognitive performance and age. The concentration of preoperative cortisol was higher among individuals with depression compared with patients without this diagnosis; however, this difference was observed only in univariate comparisons. MDD and the associated increase in HPA axis reactivity and postoperative hypercortisolemia is probably not the only pathophysiological mechanism involved in the development of postoperative delirium. For example, a higher preoperative cortisol concentration may be another contributing factor. Unfortunately, the etiology of preoperative hypercortisolemia is unknown. It may be linked to preoperative MDD or reflect other, separate and undiscovered pathologies. According to recent publications, an increased cortisol level carries a predictive value in the development of mild cognitive impairment [26]. Moreover, higher cortisol measures have also been reported in Alzheimer's disease and are associated with poorer memory performance in subjects with cognitive decline [27,28] and alterations in HPA axis activity frequently accompany aging [29].

The current analysis revealed that delirium was significantly more frequent among patients with advancing age and with lower MoCA scores. However, older age and lower MoCA scores did not maintain significance in a multivariate analysis. Older patients significantly differed from younger participants in relation to the both preoperative and postoperative cortisol concentration. Furthermore, there was a correlation between lower MoCA scores and higher preoperative cortisol level. These findings suggest that higher cortisol levels prior to surgery that act as an independent risk factor for postoperative delirium may be associated with advanced age and the impaired cognitive performance in these participants.

### Strengths and limitations

This study has several advantages. The study population was homogeneous, and the subjects were consecutive, prospectively enrolled and examined by an experienced, well-trained investigator. The analysis included a variety of factors associated with the mental and physical condition of participants, as well as those related to anesthesia and surgery. Therefore, while investigating the association between cortisol and delirium, both traumatic stress-related and psychiatric pathways were taken into consideration. To the knowledge of the authors, this represents the first study to investigate whether hypercortisolemia is a cause or an effect of delirium after cardiac surgery. This in turn allowed the analysis to control for possible confounders such as MDD and cognitive impairment, as well as factors associated with anesthesia and surgery (duration of surgery and aortic cross-clamping, dose of midazolam).

However, the study is not without limitations. The present findings cannot be considered definitive since other circulating hormones, mediators and inflammatory factors were not included in the analysis. In addition, not all prescribed medications and anesthetic agents were taken into account in the analysis, which focused on the association between the dose of midazolam and delirium. We decided to include midazolam into the analysis since this medication may decrease the level of cortisol perioperatively [30,31]. Moreover, the association between midazolam and postoperative delirium has been frequently reported [32,33]. However, the impact of other anesthetic agents that may play a role in delirium development, post-surgery sedation, as well as the impact of postoperative complications on the incidence of delirium was not assessed in this study. This being said, a recent study suggested that none of 20 different drug classes investigated (including antihypertensives, diuretics, antiplatelets and psychiatric agents) were associated with delirium after elective surgery [34].

## Conclusions

On the basis of the current analysis we can conclude that patients with raised levels of cortisol prior to surgery are at significantly increased risk of postoperative delirium. This higher level of preoperative cortisol may be associated with MDD, aging and cognitive decline.

Secondly, patients with increased HPA axis reactivity secondary to pathologies such as MDD are characterized with higher postoperative cortisol concentrations compared with patients without MDD and, possibly as a consequence, are more likely to develop postoperative delirium. These observations suggest that an increased level of cortisol may be a cause rather than an effect of postoperative delirium. Preoperative neuropsychiatric screening and monitoring of cortisol levels of cardiac surgery patients combined with postoperative surveillance may improve the early detection of delirium and, indirectly, the prognosis.

## Key messages

• Cardiac surgery patients with raised concentration of plasma cortisol prior to surgery are at significantly increased risk of postoperative delirium.

• A higher level of preoperative cortisol may be associated with MDD, advanced age and cognitive impairment.

• Preoperative diagnosis of MDD is an independent predictor of delirium after CABG surgery.

• Patients with a preoperative diagnosis of MDD have higher postoperative cortisol levels compared with patients without MDD, which may contribute to the development of delirium postoperatively.

## Abbreviations

CABG: coronary artery bypass graft; HPA: hypothalamus-pituitary-adrenal; IL: interleukin; IQR: interquartile range; MDD: major depressive disorder; MoCA: Montreal Cognitive Assessment; TMT-B: Trial Making Test Part B.

## Competing interests

The authors declare that they have no competing interests.

## Authors' contributions

JK designed the study, recruited the patients, conducted the neuropsychiatric evaluation and drafted the manuscript. AB participated in the study design and recruited the patients. JL collected and stored the patients' blood samples and the patients' data. JB drafted and revised the manuscript. RJ participated in the study design and revised the manuscript. All authors read and approved the final version of the manuscript.
